# Comparative Sublethal Toxicity of Three Neonicotinoids in Red-Winged Blackbirds

**DOI:** 10.1021/acs.est.5c03152

**Published:** 2025-07-07

**Authors:** Margaret L. Eng, Christy A. Morrissey

**Affiliations:** † Toxicology Centre, 7235 University of Saskatchewan, Saskatoon S7N 5B3, Canada; ‡ Department of Biology, University of Saskatchewan, Saskatoon S7N 5E2, Canada

**Keywords:** neonicotinoids, appetite suppression, birds, fattening index, neurotoxicity, relative toxicity

## Abstract

Seed-eating birds that use agricultural areas could be exposed to neonicotinoids through several pathways, including ingestion of treated seeds. Previous studies in birds have found sublethal exposure to neonicotinoids can reduce body mass and fat stores, but the underlying mechanism is not clear. We hypothesized that neonicotinoids reduce body mass principally by acting as an appetite suppressant rather than increasing energy consumption. Red-winged blackbirds () were exposed to an oral dose of one of three neonicotinoids at concentrations that could realistically be consumed by seed-eating birds (0, 10, 20, or 30 mg/kg bw of imidacloprid, thiamethoxam, or clothianidin) or were given a moderate or high food-restricted diet (*n* = 9 or 10 birds per treatment) and were then monitored for 3 days. Imidacloprid caused neurotoxic symptoms and rapid reductions in food consumption and body mass that persisted for >48 h. Food restriction had similar effects on body mass and biochemical indicators of energetic state (plasma triglycerides, uric acid, and β-hydroxybutyrate), which suggests appetite suppression was the main driver of mass loss in imidacloprid-exposed birds. Single doses of clothianidin and thiamethoxam altered food consumption and biochemical markers, but effects were less severe than for imidacloprid, and neither compound caused overt neurotoxicity. This study confirms that acute neonicotinoid exposures can affect behavior, appetite, mass, and energetic state in seed-eating songbirds, with potential consequences for survival.

## Graphical abstract

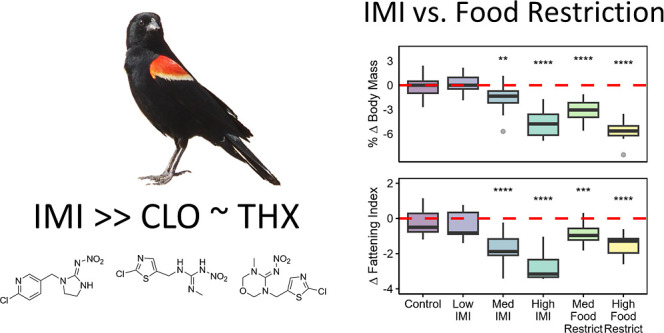

## 1 Introduction

The practice of coating seeds with insecticides prior to planting has led to a rapid and widespread increase in the use of systemic insecticides such as neonicotinoids. Neonicotinoids were introduced on the market in the mid-1990s and are now the most used insecticides worldwide as the result of a large shift in agriculture toward pre-emptive seed treatment for many crops.^1,2^ The most toxic neonicotinoids have been banned for outdoor uses in some jurisdictions, including Europe; however, neonicotinoids are still used throughout much of the world, including Canada and the US, and emergency authorizations led to their continued use in Europe post ban.^3^ Treated seeds may act as a concentrated source of exposure to neonicotinoids for seed-eating birds, and depending on the crop and application rate, the concentrations on treated seeds could be high enough to cause acutely toxic effects, including lethality, in wildlife.^4,5^ There are multiple lines of evidence that treated seeds can remain available near or above the soil surface after planting and seed spills and that birds and other wildlife will consume these seeds.^6–9^ Birds may also be exposed to neonicotinoids through contaminated water, soil, or insect prey. Although neonicotinoids are rapidly metabolized,^10–13^ there are often high detection rates (≥80%) of neonicotinoids in feces, blood, and tissues of seed-eating birds in the wild.^14–17^ Neonicotinoids are also frequently detected in birds that do not eat seeds,^18–20^ which is further evidence of their ubiquity in the environment.

Neonicotinoids have neurotoxic mechanisms of action, acting as nicotinic acetylcholine receptor (nAChR) agonists.^21^ In addition to acute lethality associated with consumption of neonicotinoid-treated seeds,^22,23^ at lower doses, neonicotinoids can also exert sublethal effects on behavior and body condition at concentrations that represent consumption of just a few treated seeds.^24–26^ One of the most overt and consistent sublethal effects observed in birds is appetite suppression and loss of body fat and mass.^27–32^ Reduced body condition is associated with negative effects for many essential processes that are linked to survival and fitness, such as immune function, growth, migration, and reproduction.^33,34^ As such, appetite suppression and subsequent mass loss could be a sublethal mechanism through which neonicotinoids could exert population-level effects.

There is evidence that reduced food consumption following neonicotinoid exposure is related to “post-ingestion gastrointestinal distress”.^35,36^ Additionally, nAChR agonists such as nicotine and neonicotinoids can alter central cholinergic-linked metabolic processes that lead to appetite suppression.^37^ It is not clear whether appetite suppression is the primary cause for reduced body mass in exposed birds or if there are other interactive toxic responses following neonicotinoid exposure that could exacerbate mass loss. For example, insecticide exposure could cause inflammatory responses that may alter digestion and nutrient assimilation efficiency, stimulate ROS-induced oxidation of lipids and proteins, increase metabolism and energy expenditure, or alter other processes that can mediate changes in body mass.

The majority of published information for neonicotinoid effects in birds is for imidacloprid (IMI),^26,28,38,39^ and less is known about how other major neonicotinoids, such as clothianidin (CLO) and thiamethoxam (THX), that have become more widely used affect birds at sublethal concentrations. Available evidence indicates that IMI is the most toxic for birds and other vertebrate wildlife.^4^ A study in eared doves () found that IMI is 70× more acutely toxic than THX or CLO.^31^ However, there can be large differences in relative toxicity and species sensitivity to contaminants. For example, in invertebrates, meta-analyses and multispecies assessments indicate that THX is generally less toxic than IMI or CLO and that there can be several orders of magnitude difference in species sensitivity.^40,41^ At the species level, there is variation in relative toxicity, and in some species, THX is more toxic than CLO or IMI.^40^ To date, there have not been any direct toxicity comparisons of different neonicotinoid active ingredients in seed-eating songbirds.

The objectives of this study were to (1) compare the relative toxicity of three major neonicotinoids (IMI, CLO, and THX) on food consumption, body mass, and signs of neurotoxicity in a seed-eating songbird and (2) to evaluate whether the evidence of appetite suppression and reduced food consumption or other confounding physiological mechanisms are the primary cause of observed mass loss. Red-winged blackbirds () were selected as a study species, as they are common and robust seed-eating farmland songbird species that have a large enough body size (∼40–70 g) to allow for repeated blood sampling and are amenable to captivity. While they are common and widespread, they also exhibit population declines across North America.^42^ Blackbirds were orally dosed with a vehicle control or one of three different doses of IMI, CLO, or THX that were at field-relevant concentrations and predicted to be sublethal. Two additional groups were food restricted at moderate and high levels to simulate appetite suppression and evaluate the physiological effect of reduced food consumption. Blood samples from all groups were analyzed for plasma metabolites (triglycerides, β-hydroxybutyrate [β-OHB], and uric acid), which are sensitive indicators of metabolism and physiological states such as fat deposition, protein catabolism, and fasting.^43^

## 2 Materials and Methods

### 2.1 Study Species

Red-winged blackbirds (*n* = 125) were captured prior to the breeding season over a period of 12 days in May of 2018 at a wetland complex southeast of Saskatoon, Saskatchewan (52° 2′ N, 106° 32′ W), and transported to the Facility for Applied Avian Research at the University of Saskatchewan, where they were held as smaller groups (*n* ∼ 20) in outdoor aviaries (4 m L × 2.5 m W × 2.5 m H) until the start of the experiment. Birds were initially fed an ad libitum diet of black oil sunflower seeds, millet, and poultry starter crumbles (Proform 26%). At the start of the dosing experiment, birds were transferred to individual cages (61 cm L ×  61 cm W  ×  61 cm H) to allow for easier handling and monitoring and acclimatized to cages for 4 days, at which point body mass was stable in all birds. Experimental birds were provided exclusively organic turkey poultry starter (Country Junction 17% Backyard Poultry Grower) to allow for ease of measuring due to lack of husks and filtered water ad libitum. All research protocols were in compliance with the Canadian Council on Animal Care guidelines and approved by the University of Saskatchewan Animal Care Committee (AUP 20110043). At the end of the experiment, birds were examined by the university research veterinarian and were released at the site of capture if judged to be fit and healthy.

### 2.2 Experimental Procedures

For each compound (IMI, CLO, and THX), 10 birds were randomly assigned to one of three treatment groups (low, medium, and high) and fed ad libitum. All treatment groups had both male and female birds. Final sample sizes for IMI low and CLO high were 9, due to removal of birds from the study for reasons unrelated to treatment. In addition, 9 birds per group were randomly assigned to either moderate or high food restriction (food availability reduced to 71% or 38% of typical daily consumption, respectively), and 15 birds were assigned to the vehicle control (sunflower oil) group and fed ad libitum. Dosing volume was 10 μL/g body mass, and nominal doses of all compounds were 10, 20, or 30 mg/kg body weight (bw) for the low, medium, and high treatment groups, respectively. Dosing was a single acute exposure through oral gavage using 20G curved stainless steel crop needles attached to disposable syringes. Birds were all dosed between 09:00 and 11:00.

Birds in all treatment groups were monitored for clinical signs of neurotoxicity at frequent intervals (<5 min) for the first hour following gavage to record the time of first symptoms. At approximately 1, 6, and 24 h postdosing, all birds were scored for severity of neurobehavioral symptoms. Neurobehavioral severity scores were based on activity levels, balance/ataxia, fluffed feathers, responsiveness, and respiratory effort (Table S1). Birds were assigned a severity score between 0 (normal, active) and 5 (virtual paralysis). As behavioral observers had also conducted the dosing, observers were not completely blind to treatment; however, treatments were randomly assigned to cages throughout the pen, and there were no visual indicators on the cages to identify treatment, dose groups were not identified at the time of behavioral scoring, and behavioral scores were based on well-defined criteria that could be objectively judged.

Pre- and postdosing blood samples (∼100 μL) were taken at the same time on consecutive days. Predosing samples were taken between 15:00 and 17:00 the day before dosing, and postdosing samples were taken between 15:00 and 17:00 the day of dosing (i.e., at 6 h postdosing). All blood samples were collected in heparinized capillary tubes following venipuncture of the brachial vein with a 26G needle. Blood samples were centrifuged within 1 h of collection, and plasma was separated from red blood cells and stored frozen at −80 °C until analysis.

Food was weighed daily between 9:00 and 11:00 (24 h before dosing, at the same time as dosing, and at 24 and 48 h after dosing). Unconsumed food, including food remaining in the bowl as well as any spilled food, was collected and weighed and subtracted from the initial mass of food provided to determine daily food consumption (predosing, first 24 h after dosing, and 24–48 h after dosing). A sample food dish placed outside of the cages was measured daily to account for any change in food mass due to desiccation or moisture. For the duration of the experiment, we did not measure any detectable (>0.1 g) changes in the mass of the external food. Daily food consumed was divided by daily body mass to calculate food consumption on a per body mass basis. Food consumption could not be calculated for 4 birds (*n* = 2 CLO high, *n* = 2 CLO low) due to excess spillage and soiling of food in cages.

Food restriction trials were designed to replicate average daily food consumption following exposure to the medium or high dose of IMI. On the experimental food restriction day, each bird in the “moderate” food restriction group was given 0.25 g food/g body mass, which is equivalent to the average food consumption in the medium IMI dose birds in the 24 h period following dosing. Birds in the “high” food restriction group were each given 0.13 g food/g body mass, which is equivalent to the average food consumption in the high IMI dose birds in the first 24 h postdosing. In comparison, the average pretreatment food consumption across all treatment groups was 0.34 g food/g body mass/day; therefore, the available food for the moderate and high food-restricted groups, respectively, represents 71% (0.25 g/0.34 g) and 38% (0.13 g/0.34 g) of typical food consumption. Birds were housed individually in cages for the food restriction trials and were acclimated to cages for 5 days until body mass was stable before food restriction trials began. During the trials, between 09:00 and 10:00, each bird was weighed and provided with the equivalent of one-third of their daily food allowance. Then, 6 h later (between 15:00 and 16:00, immediately following blood sampling), the remaining two-thirds of their daily food was provided. Food was dispensed incrementally at these two time points to prevent food-restricted birds from consuming the full portion of food within the first 6 h and so that blood samples accurately reflected restricted food intake. If any food was remaining, it was also weighed at each time point to calculate actual food consumption. Birds were returned to their regular ad libitum diet at 09:00 the following day (24 h after initiation of food restriction), and body mass and food consumption were weighed between 09:00 and 10:00 for three days to monitor recovery. Birds were blood sampled between 15:00 and 16:00 the day before food restriction and again between 15:00 and 16:00 the day of food restriction (i.e., 6 h). Timing of blood sampling and weighing corresponded to the same timeline used in the neonicotinoid dosing trials.

### 2.3 Plasma Metabolite Analysis

For all plasma metabolite analyses, individual blackbird plasma samples were run in duplicate, and an in-house European starling () plasma pool was included as a reference material in all 7 plates for each assay to assess interassay variation. Plasma triglyceride concentration (mmol L^–1^) was measured using 5 μL plasma per well in a sequential colorimetric endpoint assay for free glycerol and total triglyceride (Sigma F6428, T2449, and G7793), with plasma triglyceride calculated as the difference between the two. Average intra-assay CV (based on duplicate samples) for plasma triglycerides was 1.7%, and average interassay CV was 8.9%. β-OHB (mmol L^–1^) was measured using a colorimetric assay (Cayman 700,190-96) following kit instructions, with samples diluted 15× in assay buffer. Average β-OHB intra-assay CV was 2.4%, and average interassay CV was 3.2%. Uric acid (mmol L^–1^) was measured using a colorimetric assay (Thermofisher A22181) following kit instructions, with samples diluted 20× in reaction buffer. Average uric acid intra-assay CV was 2.6%, and average interassay CV was 1.8%.

### 2.4 Dosing Solutions

Dosing solutions were prepared and analyzed using direct extraction following the same methods and instrumentation as described for the dosing solutions in Eng et al.^28^ Dosing solutions were prepared by dissolving technical-grade IMI, CLO, or THX (Sigma-Aldrich 37,894, 33,589, and 37,924), in a small volume of acetone, then diluting with organic sunflower oil (Compliments brand, Sobeys Canada), and then left to stir overnight to evaporate off the acetone and stored in glass vials protected from light for the duration of the study. Control oil was treated the same without the addition of neonicotinoids. Solutions were analyzed at the National Hydrology Research Centre, Environment and Climate Change Canada, Saskatoon, SK, using liquid chromatography–tandem mass spectrometry (LC–MS/MS). Solutions were first diluted 100× into an intermediate solvent (acetone) and then further diluted with water to within the calibration range (50×, 200×, 400×, and 667× for control, low, medium, and high solutions, respectively). Diluted aqueous solutions were directly injected into the mass spectrometer (Waters 2695 Alliance HPLC system; Waters Corp., Milford, MA). The instrument limits of detection (LODs) were 0.53, 0.63, 1.61, and 0.35 ng/mL for IMI, CLO, THX, and ACE, respectively. The limits of quantification (LOQs) were 1.59, 1.90, 4.84, and 1.05 ng/mL for IMI, CLO, THX, and ACE, respectively.

Nominal dosing solution concentrations were 1, 2, or 3 mg/mL based on a dosing volume of 10 μL/g body mass and a target dose of 10, 20, or 30 mg/kg bw for the low, medium, and high treatment groups, respectively. Measured dosing solution concentrations were within the acceptable range of error: 0.69, 2.00, and 3.35 mg/mL for IMI; 0.85, 2.08, and 3.13 mg/mL for CLO; and 0.94, 1.45, and 3.08 mg/mL for THX. There were no detectable neonicotinoids (IMI, CLO, THX, and ACE) in the vehicle control solution, and dosing solutions also did not have detectable background contamination from any other neonicotinoids.

### 2.5 Statistical Analysis

Statistical analysis was completed using SAS 9.4 and R 4.2.3. Time-to-event analysis to determine median time from dosing in minutes to first symptoms appearing was analyzed using the “survival” package in R, using the surv­() function for right-censored data fit to the Kaplan–Meier curve. Censoring status was coded as 0 (no symptom event) or 1 (symptoms). The effect of treatment on neurotoxicity scores was assessed using the Kruskal–Wallis test, and for significant effects, differences between treatment groups were assessed using Dwass, Steel, Critchlow–Fligner (DSCF) multiple comparison analysis. Plasma triglyceride and β-OHB are typically negatively correlated with each other, and principal component analysis (PCA) can be used to derive a single “fattening index” based on the concentrations of the two metabolites.^44,45^ Comparisons of mass, daily food consumption, uric acid, and fattening index between treatments (control, IMI low, IMI med, IMI high, CLO low, CLO med, CLO high, THX low, THX med, THX high, moderate food restriction, high food restriction) over time were made using general linear mixed models (proc MIXED), with bird ID as a repeated subject effect and fixed effects of treatment, time, treatment*time, and sex. For the “time” variable, mass and daily food consumption were compared immediately predosing, 24 h postdosing, and 48 h postdosing. For plasma metabolites, predosing and postdosing samples that were taken 24 h apart were compared. Percent change in consumption or body mass was calculated using the equation [(×2 – ×1)/×1]*100, where ×2 represents the final value (at either 24 or 28 h) and ×1 the initial value (predosing). For significant effects, least-squares means were contrasted to compare predosing measures to postdosing measures within treatment groups. Model assumptions were examined visually by plotting model residuals against predicted values and with a residual quantile–quantile plot. Daily food consumption, body mass, plasma triglycerides, and β-OHB were log transformed so that model residuals met the assumptions of normality and heteroscedasticity. All significance levels were set at α = 0.05.

## 3 Results and Discussion

There were significant effects of neonicotinoid treatment on behavioral signs of neurotoxicity, food consumption, body mass, and physiological state in red-winged blackbirds following a single oral exposure. Control birds did not exhibit significant changes in any endpoints over time, while IMI exerted the strongest negative effects on all measured endpoints, and most observed effects were dose-dependent. CLO and THX had fewer and less severe effects than IMI at the same exposure concentrations. CLO altered food consumption and the fattening index, and THX altered food consumption, body mass, and uric acid; however, neither compound caused overt behavioral neurotoxicity symptoms.

The doses used in this study are in the range of concentrations that a midsize bird could be exposed to if it were to consume treated seeds when foraging in an agricultural field. The amount of active ingredient (a.i.) on treated seeds will vary by crop and commercial product, but for example, US label application rates for CLO (Poncho 600) are up to 1.25 mg a.i./corn seed, and for IMI (Gaucho 600) are 1.34 mg a.i./corn seed and 0.93 mg a.i./g wheat seed (2.4 fl oz of product per 100 lb of seed with 5 lb of IMI per gallon of product). THX (Helix Vibrance) has application rates of 4 mg a.i./g for canola seed (23 fl oz of product per 100 lb of seed, with 2.25 lb of THX per gallon of product). The average predosing body mass of birds in our study was 60.6 g, so a dose of 10 mg/kg bw would be equal to ingestion of 0.6 mg total a.i. Therefore, the low dose in our study would be equivalent to consumption of less than one corn kernel or less than a gram of seed for a midsized songbird. For reference, pretreatment birds in our study consumed, on average, 19 g of untreated food per day.

### 3.1 Neurotoxicity

IMI exerted significant acute neurotoxic effects on red-winged blackbirds in our study, while CLO and THX exposure did not result in any observable signs of neurotoxicity, and no control birds displayed signs of neurotoxicity. Neurobehavioral toxicity of IMI has been observed for several species of birds, including documented instances of field poisoning. Cape spurfowl () that consumed IMI-treated cereal seed in farm fields exhibited loss of coordination and ability to fly, with some birds suffering fatality.^23^ Out of 101 identified IMI poisoning incidents from a national pesticide surveillance program in France, 39.6% of the incidents described neurobehavioral effects (sudden fall in flight, ataxia, paralysis or paresis, behavior disturbance, disorientation, impaired alertness, apathy) across six different avian species.^22^ Low concentrations (1 or 6 mg/kg bw) of IMI caused hyperreactivity to simulated predator threats in red-legged partridges.^46^ In contrast, neurobehavioral effects of CLO and THX are not commonly reported. In a captive study of South American eared doves, IMI caused severe intoxication (motionless, unresponsive, unable to sit) at the lowest tested dose (14.1 mg/kg bw), while severe symptoms were not observed until doses of 1281 mg/kg bw for CLO and 800 mg/kg bw for THX.^31^ Those concentrations of CLO and THX are 1 to 2 orders of magnitude higher than the ones used in our study, and the doses we used were likely not high enough to cause overt neurobehavioral effects.

In red-winged blackbirds that exhibited neurotoxic symptoms following IMI exposure, we observed the first symptoms between 3 and 106 min, with all but one bird showing symptoms within 40 min of dosing (Figure 1A). Timing of the onset of symptoms decreased with increasing dose. The estimated median time to first symptoms was 9 min (range 3–29) in the high dose, and all birds in the group exhibited symptoms (*n* = 10). In the medium dose, 90% of birds (*n* = 9/10) showed symptoms, with a median time of 24.5 min (range 6–106). The median time was not estimated for the low dose group as less than half of the birds (*n* = 4/9) exhibited symptoms, but in those that did show symptoms, they were observed between 10 and 36 min after dosing.

**Figure 1:**
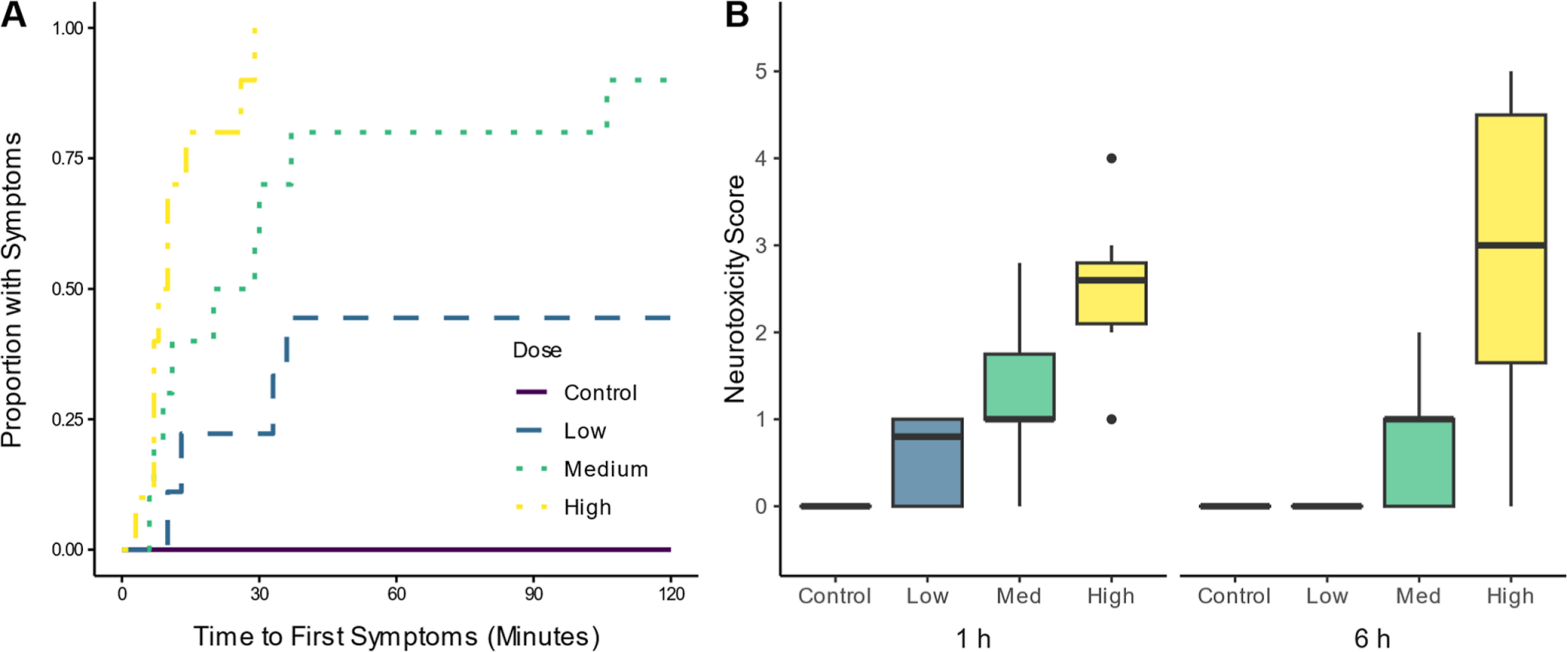
Cumulative proportion of captive red-winged blackbirds exposed to a vehicle control (sunflower oil) or low, medium, or high doses (10, 20, or 30 mg/kg bw) of imidacloprid exhibiting symptoms over time from dosing (A) and the scores for severity of symptoms based on neurotoxicity evaluations at 1 h and 6 h postdosing (B). Neurobehavioral abnormalities were scored from 0 (normal, active) to 5 (virtual paralysis). Boxes indicate interquartile range (IQR), middle lines indicate median, whiskers show the minimum and maximum values within 1.5× IQR, and circles represent outliers (>1.5× IQR from box).

At 1 h postexposure, we found a significant difference between IMI-exposed groups in the severity of symptoms based on neurotoxicity scores (χ^2^_3_ = 34.2, *p* < 0.0001; Figure 1B). All control birds had a score of zero. The median neurotoxicity scores for the low IMI and medium IMI dose groups were significantly lower than for the high IMI dose group, and all birds in the high dose group exhibited some signs of neurotoxicity (median low score = 0.8, range = 0.0–1.0; medium score = 1.0, range 0.0–2.8; high score = 2.6, range 1.0–4.0). By 6 h postexposure, there were still differences in neurotoxicity symptoms between treatment groups (χ^2^_3_ = 26.4, *p* < 0.0001). All low dose birds had fully recovered within 6 h with neurotoxicity scores of zero. However, the median neurotoxicity score in the medium IMI group was still elevated at 6 h with similar scores to 1 h postexposure (score = 1.0, range 0.0–2.0). Signs of neurotoxicity worsened overall in the high dose group at 6 h postdosing compared to 1 h postdosing, although some birds recovered (median score = 3.0, range 0.0–5.0). Two birds (20%) in the high IMI dose group died, one within 6 h postexposure and one within 24 h postexposure.

All surviving birds recovered within 24 h, and there were no continued visible signs of neurotoxicity. This pattern of rapid neurobehavioral toxicity and recovery in nonfatal exposures is similar to what has been observed in other avian studies. For example, domestic chickens () that received 7 consecutive daily doses of IMI exhibited mild neurotoxic symptoms at 3.42 mg/kg/d and severe neurotoxicity at 15.5 mg/kg/d, with the onset of symptoms typically less than 30 min postexposure. Chickens recovered between repeat exposures, and there did not appear to be cumulative effects.^24^ In South American eared doves exposed to a wide range of IMI concentrations (14.1 to 1075.0 mg/kg bw), the onset of symptoms was typically within an hour postexposure. For exposures that were not lethal, recovery times ranged from 4.5 to 51 h, and birds dosed with less than 40 mg/kg bw all recovered within 24 h.^31^ A study in another passerine, grayish baywings (), exposed to IMI at doses ranging from 8.48 to 424 mg/kg bw also observed dose-dependent signs of intoxication, with symptoms appearing within 30 min. Mild symptoms started at 20.6 mg/kg bw, severe symptoms at 35 mg/kg bw, and all surviving birds recovered within 48 h.^5^ While recovery is possible when acute exposure does not cause mortality, in free-living birds, insecticide-induced reductions in foraging ability, responsiveness, and coordination could result in reduced body condition and increased susceptibility to predation, weather, and other stressors affecting survival.^46^

### 3.2 Food Consumption and Body Mass

In red-winged blackbirds, food consumption was significantly affected by treatment (treatment*time interaction *F*_22,208_ = 31.18, *p* < 0.0001). For control birds, daily food consumption did not significantly change compared to predosing levels in the first 24 h (−1.3%, *p* = 0.57) or 48 h (+9.6%, *p* = 0.15) after dosing. In contrast, birds exposed to IMI showed a significant dose-dependent reduction in daily food consumption of −68.6% high IMI (*p* < 0.0001), −25.1% medium IMI (*p* < 0.0001), and −17.1% low IMI (*p* = 0.0006) in the first 24 h postexposure compared to predosing (Figure 2A). High- and low dose IMI birds did not fully recover appetite by 48 h postdosing compared to predosing (high IMI: −17.4%, *p* = 0.02; low IMI: −18.6%, *p* = 0.002; Table S2, Figure S1), while daily food consumption in medium dose birds did fully recover to equivalent predosing levels (+13.7%, *p* = 0.10). The effects of CLO on food consumption were not as drastic as for IMI, although there was a reduction in daily food consumption in the first 24 h postdosing (−15.6%, *p* = 0.02) in the high dose CLO birds, which then recovered by 48 h postdosing (+15.9%, *p* = 0.15). The medium CLO dose group’s daily food consumption did not significantly change over time (24 h post, −5.1%, *p* = 0.42; 48 h post, +10.4%, *p* = 0.17). Daily food consumption in the low CLO dose group did not significantly change in the first 24 h postdosing (−8.6%, *p* = 0.24) but increased between 24 and 48 h postdosing compared to predosing levels (+17.2%, *p* = 0.03). For THX, there was a decrease in daily food consumption in the medium- (−13.5%, *p* = 0.03) and low dose groups (−16.0%, *p* = 0.007), but not in the high dose group, in the first 24 h postexposure. By 48 h postexposure, there were no differences in food consumption compared to predosing levels for any THX treatment group (*p* ≥ 0.29).

**Figure 2:**
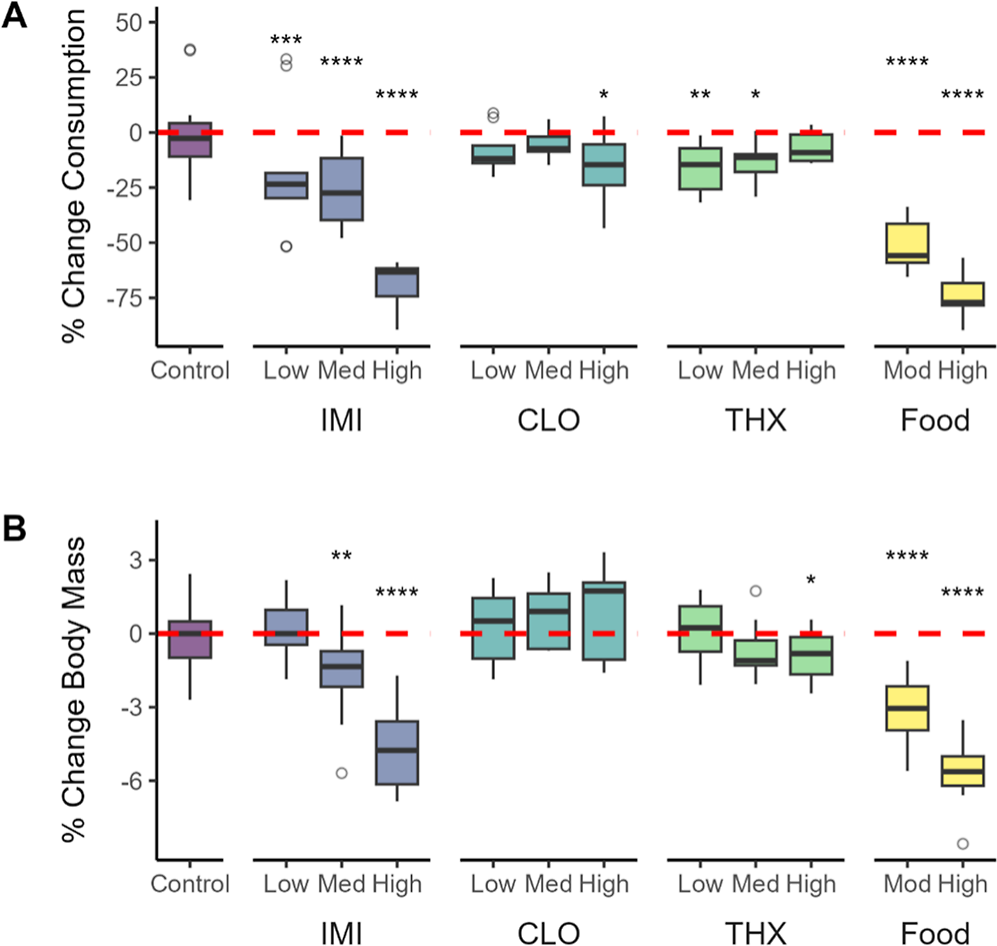
Percent change in (A) daily food consumption and (B) body mass in red-winged blackbirds exposed to a vehicle control (sunflower oil), imidacloprid (IMI), clothianidin (CLO), thiamethoxam (THX), or food restriction (moderate or high), relative to predosing levels. Changes are presented for measures taken 24 h postdosing compared to immediately prior to dosing, with the red dashed line representing no change. Asterisks indicate significant differences between predosing and postdosing measures within a treatment group (*p < 0.05, **p < 0.01, ***p < 0.001, ****p < 0.0001). Boxes indicate interquartile range (IQR), middle lines indicate median, whiskers show the minimum and maximum values within 1.5× IQR, and circles represent outliers (>1.5× IQR from box).

Food-restricted treatments were successful in experimentally lowering total food consumption. In the highly restricted group, daily food consumption significantly decreased compared to pretreatment levels (−73.8%, *p* < 0.0001). Birds ate almost all of the provided food (provided 0.13 g food/g bw/day, average consumed was 0.12 g/g bw/day for females and 0.13 g/g bw/day for males; Table S2), and the change in consumption was similar to the change in consumption observed in the high IMI treatment group (−68.6%). In the moderate food restriction group, daily food consumption was also significantly reduced during treatment (−52.4%), *p* < 0.0001), although to a greater extent than in the medium IMI group (−25.1%). Male birds in this group did not eat the full amount of food provided (provided 0.25 g food/g bw, average consumed was 0.25 g/g bw/day for females and 0.17 g/g bw/day for males). To more closely match food consumption with moderate food restrictions, it is possible that slightly more food than the target consumption needs to be provided. Both food-restricted groups fully recovered to pretreatment daily food consumption rates within the first 24 h of returning to ad libitum food (+12.1%, *p* = 0.11 and +6.9%, *p* = 0.35 in high and moderate food-restricted groups, respectively), suggesting there are no sustained effects of food restriction on appetite. This contrasts with IMI exposure, which continued to reduce food consumption 48 h after exposure in the low- and high-dose groups (Figure S1). The persistence of anorexic effects of imidacloprid for multiple days postexposure has also been observed in eared doves^32^

Body mass showed a dose–response relationship, with a significant interaction between treatment and time (*F*_22,214_ = 10.12, *p* < 0.0001). Body mass in the control birds did not significantly change from predosing to 24 h postdosing (*p* = 0.666) or 48 h postdosing (*p* = 0.41), whereas IMI exposure caused significant body mass loss in high dose (−4.6% body mass, *p* < 0.0001) and medium dose (−1.6% body mass, *p* = 0.001) birds at 24 h postexposure (Figure 2B and Table S2). By 48 h postexposure, medium IMI dosed birds recovered to their predosing mass (*p* = 0.13), but high IMI dosed birds had not fully recovered (−1.3% compared to predosing mass, *p* = 0.019). Although low IMI dose birds reduced food consumption, body mass remained stable, and there was no significant difference in body mass at 24 h postdosing compared to predosing (*p* = 0.83), followed by a mass gain by 48 h postdosing (+1.3% body mass, *p* = 0.02).

In contrast to IMI, CLO exposure did not have any effects on body mass over time for any dose group (*p* > 0.10), and the effects of THX on body mass were more subtle. There was a nonsignificant dose-dependent trend of decreasing body mass in the first 24 h postexposure (high THX dose decreased 0.93% [*p* = 0.05], medium THX dose decreased 0.70% [*p* = 0.14], and low THX dose stayed the same [+0.11%, *p* = 0.82]). Mass then increased slightly during the recovery period, and by 48 h postexposure, body mass in the high THX dose birds returned to predosing levels (*p* = 0.59), medium THX dose birds had a nonsignificant increase (+0.84%, *p* = 0.09), and low THX dose birds had a significant increase (+1.0%, *p* = 0.044) in body mass compared to predosing mass.

In food-restricted birds, we did find clear confirmation that a reduction in food consumption can cause a rapid loss of body mass. Body mass was significantly lower 24 h after food restriction started compared to before food restriction (high food restriction, −5.7%; moderate food restriction, −2.4% body mass; *p* < 0.0001). This decrease in body mass was comparable to the IMI high- and medium-dose treatment groups. At 24 h after birds were returned to ad libitum food (i.e., 48 h after food restriction started), birds in both the high and moderate food restriction groups had not fully recovered to their normal body mass (−2.4%, *p* < −0.0001, and −1.0%, *p* = 0.05, respectively).

The effect of treatment on food consumption and body mass was not affected by sex (treatment*sex interaction *p* > 0.05), and the interaction term was not included in the final model. Males were significantly heavier than females (*F*_1,108_ = 1764.00, *p* < 0.0001), and females consumed more food per gram of body mass (*F*_1,106_ = 77.44, *p* < 0.0001), so sex was included in all models to control for these effects. Food consumption appears to be a slightly more sensitive end point than body mass, as in some treatment groups (e.g., low IMI, high CLO), food consumption but not mass was significant. It is possible that the change in consumption rate in these dose groups was not large enough to have a measurable effect on body mass. Additionally, higher body mass does not necessarily reflect improved condition; for example, Japanese quail () exposed to 50 mg CLO/kg exhibited body weight gain that was attributed to impaired hepatic function leading to enlarged livers.^47^

### 3.3 Plasma Metabolites

Plasma triglycerides and β-OHB are reliable indicators of the metabolic state of songbirds (fueling or fasting).^44,45,48^ Triglycerides are composed of three fatty acids and a glycerol backbone. During feeding, there is increased synthesis of triglycerides by the liver, and elevated plasma triglycerides are associated with fueling and mass gain.^49^ β-OHB is a ketone body synthesized by the liver during fat catabolism, and β-OHB levels increase during fasting.^49^ Plasma triglycerides and β-OHB are typically negatively correlated with each other, which is what we observed in red-winged blackbirds (*r* = −0.25, *p* = 0.0001). Using PCA to infer the “fattening index”, the first principal component of plasma triglycerides and β-OHB explained 62.4% of the variance and was positively associated with plasma triglycerides (fueling) and negatively associated with β-OHB (fasting).

Treatment significantly affected how the fattening index changed from predosing compared to 6 h postdosing (treatment*time interaction *F*_11,107_ = 10.82, *p* < 0.0001). In the control group, the fattening index did not change over time (*p* = 0.21), whereas the fattening index significantly decreased in both the high IMI dose (*p* < 0.0001) and medium IMI dose (*p* < 0.0001) groups, but did not significantly change in the low IMI dose group (*p* = 0.11) (Figure 3A; Table S3). For CLO, the fattening index was lower postdosing in both the high dose (*p* = 0.03) and medium dose (*p* = 0.006) groups but not the low dose group (*p* = 0.14). For THX, there were no differences between the pre- and postdose fattening index for any dose group (*p* ≥ = 0.34). We observed significant decreases in the fattening index in both the high (*p* < 0.0001) and medium (*p* = 0.0004) food-restricted groups, which were comparable to those observed in the high and medium IMI dose treatment groups.

**Figure 3:**
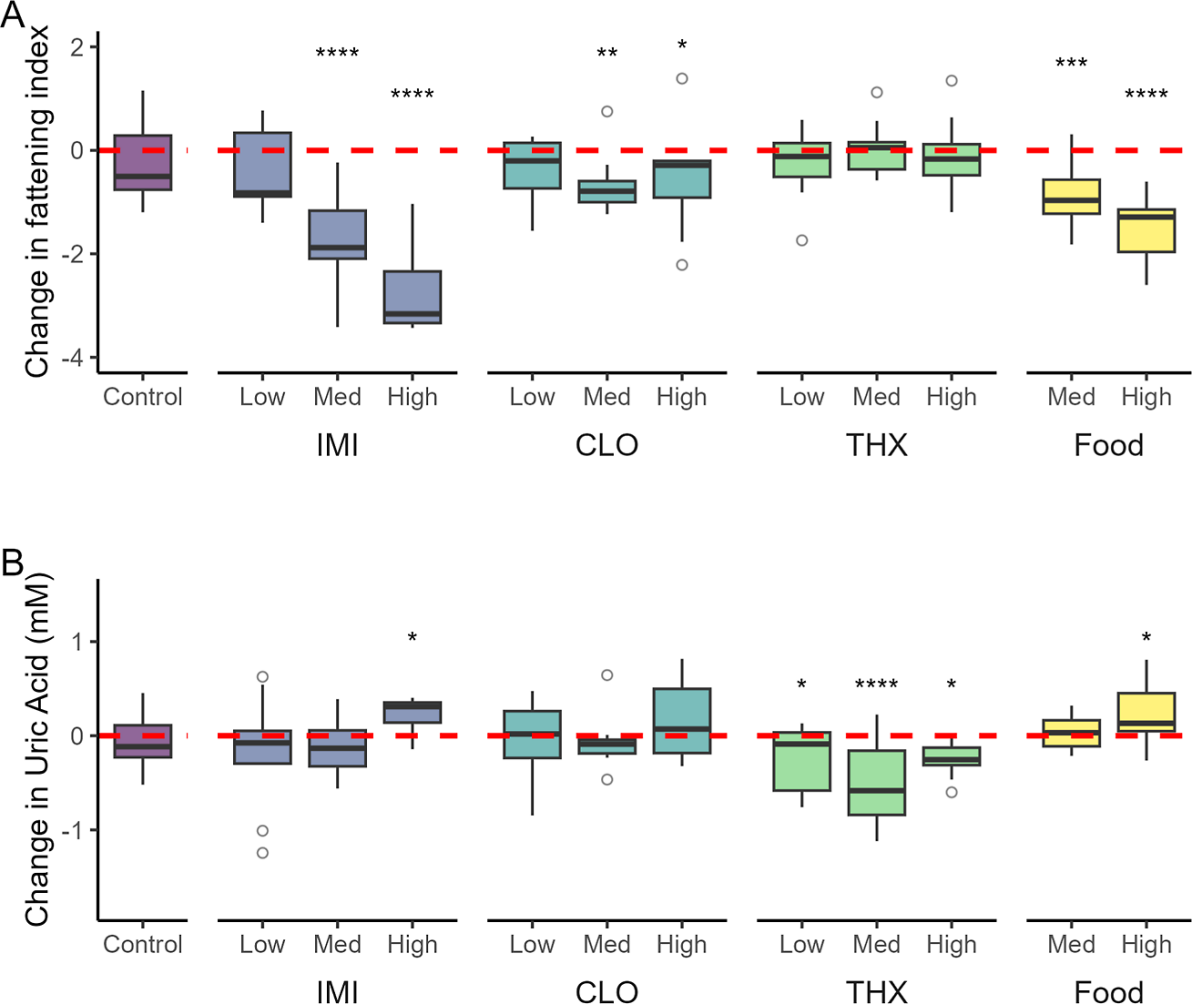
Change in (A) fattening index and (B) uric acid in red-winged blackbirds exposed to a vehicle control (sunflower oil), imidacloprid (IMI), clothianidin (CLO), thiamethoxam (THX), or restricted food (moderate or high). Changes are presented for the difference between blood samples taken at 6 h postdosing compared to blood samples taken at the same time the previous day (18 h predosing), with the red dashed line representing no change. Asterisks indicate significant differences between predosing and postdosing measures within a treatment group (*p < 0.05, **p < 0.01, ***p < 0.001, ****p < 0.0001). Boxes indicate interquartile range (IQR), middle lines indicate median, whiskers show the minimum and maximum values within 1.5× IQR, and circles represent outliers (>1.5× IQR from box).

Uric acid is an indicator of protein catabolism, which can be elevated either during feeding from the use of dietary protein or during starvation from catabolism of stored protein, whereas it typically decreases during minor or routine fasting periods (e.g., overnight).^50–52^ The change in uric acid between pre- and postdosing was significantly affected by treatment (treatment*time *F*_11,107_ = 3.49, *p* = 0.0003). There was no change of uric acid over time in the control group (*p* = 0.408), while in birds exposed to IMI, uric acid increased in the high group (*p* = 0.03) but did not change in the medium dose (*p* = 0.32) or low dose group (*p* = 0.12) (Figure 3B). There was no change in uric acid for any of the CLO exposure groups (*p* ≥ 0.13), and uric acid decreased in the high (*p* = 0.03), medium (*p* < 0.0001), and low THX dose groups (*p* = 0.03). In the high food restriction group, uric acid significantly increased (*p* = 0.05), but uric acid did not significantly change in the medium food restriction group (*p* = 0.76). There was no interactive effect of treatment and sex on the fattening index (*F*_11,97_ = 1.18, *p* = 0.31), and there was no effect of sex on the fattening index (*F*_1,108_ = 0.94, *p* = 0.33). Similarly, there was no interactive effect of treatment and sex on uric acid (*F*_11,97_ = 0.72, *p* = 0.72) or of sex on uric acid (*F*_1,108_ = 0.60, *p* = 0.44), so sex was not included in the final models for plasma metabolite analyses.

### 3.4 Neonicotinoids as “Anorexic Agents”

Collectively, the large decrease in fattening index observed in the IMI-dosed and food-restricted birds corresponds with the large decreases in food consumption and body mass observed in the same treatment groups, suggesting that neonicotinoids can exert “anorexic” effects. The fattening index, food consumption, and body mass relationships in the CLO and TMX groups were less clear. High CLO had a lower fattening index and food consumption but a nonsignificant trend for increased body mass. The medium CLO group had a lower fattening index but no effect on consumption or body mass. The increase in uric acid in high IMI and high food restriction birds likely reflects a state of fasting and initiation of protein catabolism, whereas the decrease in uric acid in the THX treatments suggests low protein utilization and may indicate moderate fasting that is not outside of the normal physiological range, such as when uric acid levels decrease during overnight fasting in songbirds.^50^ This corresponds with the minor reductions in food consumption and body mass that were observed in THX-exposed birds. It is possible that the fattening index is a more sensitive indicator of effect than food consumption or body mass, or that CLO or TMX exposure could be interacting with body mass through mechanisms other than reduced food consumption (e.g., enlarged organ weights). More detailed assessments, such as body composition analysis (fat, lean mass, water) and organ histology, could provide more information on more subtle changes in physiology following neonicotinoid exposure. Plasma metabolites were measured at approximately the same time in the late afternoon each day, when birds had access to ad libitum food (except for food-restricted birds during the first 24 h), so time of day would not explain these results.

The similar mass loss and metabolite patterns that were observed in IMI dosed birds compared to food-restricted birds suggest that the main mechanism of reduced body condition following acute exposure to sublethal IMI concentrations is likely due to appetite suppression leading to reduced food consumption. Neonicotinoid exposure in birds appears to consistently cause similar anorexic symptoms of lower appetite, lower food consumption, and loss of body mass across species.^53^ For example, white-crowned sparrows () dosed by gavage at 4.1 mg/kg bw showed symptoms of ataxia and lethargy, lower food consumption, and up to a 25% reduction in body mass after 3 days.^28^ In another study of white-crowned sparrows, birds exposed to a single dose of 3.9 mg/kg reduced food consumption by 70% compared to controls and lost 5.9% of their body mass and 17.1% of their body fat within 6 h of exposure.^29^ South American eared doves exposed to higher doses of IMI (up to 90 mg/kg), CLO, or THX (>2000 mg/kg) showed dramatic reductions in food consumption and body mass.^31^ In passerine grayish baywings fed IMI-treated seed at average application rates (3 g IMI/kg seed), food intake rate drastically decreased by 74 to 90% compared to pre-exposure food consumption.^5^ When feed seeds were treated with just 15% of average application rates (0.45 g IMI/kg seed), grayish baywings reduced food consumption by 20% in the first 3 days of exposure, and food consumption then fluctuated over a 32 day period, while body mass decreased by 8% in the first week of exposure and remained reduced for the duration of the experiment.^54^ An unpublished Bayer (2016) report (obtained through a request from Health Canada’s PMRA doc# 2,744,282) revealed a synthesis of findings from 33 industry avian feeding trials conducted over 2 decades involving various species of captive granivorous birds exposed to choices of IMI- or CLO-treated and untreated seed. In 20 of 33 (60%) trials, researchers reported loss of body mass, and many birds showed signs of “post-ingestion distress” and overt signs of intoxication. In mammals, reductions in body mass have similarly been observed when exposed to high dietary concentrations of neonicotinoids, which is thought to be a generalized, nonspecific effect associated with reduced food consumption.^37,55^

The observed reduction in food consumption and mass in animals following neonicotinoid exposure does not appear to be solely due to treated seed repellency.^35^ In several of the studies, including the current study in red-winged blackbirds, birds were dosed directly via gavage, and all feed seed was untreated. It is possible that birds could have negative associations with the food type that was being eaten when experiencing intoxication. However, in our study, birds were being provided with the same food before and after dosing, and it is not likely that this was the case. Alternatively, neonicotinoids could directly cause reduced appetite. Further studies that introduce novel foods post-treatment would be needed to distinguish the influence of food type association on consumption. Neonicotinoids are an nAChR agonist and are structurally similar to nicotine, which has known appetite-suppressing properties in human and animal models.^56^ Extensive research confirms that nicotine’s effect on appetite and food consumption is through complex agonism of nAChRs present in both neural (e.g., hypothalamus and nucleus of the tractus solitarius) and non-neural tissues (gastrointestinal tract, liver, pancreas, adipocytes).^37^ Neonicotinoid detoxification can also induce higher energy expenditure and metabolism, leading to reductions in body mass.^57^ However, in more metabolically active species such as ruby-throated hummingbirds (), which have very high energetic demands, exposure to low doses of IMI (1 to 2.5 mg IMI/kg bw per day for 3 days) can cause decreased energy expenditure.^39^ There is a growing body of evidence that acute neonicotinoid exposures to IMI, and to a lesser extent CLO and THX, have mechanisms that exert sublethal effects on appetite, food consumption, and body mass across many species, which are critically important fitness determinants in the wild.

### 3.5 Relative Toxicity

For each endpoint, we assessed (neurotoxicity, food consumption, body mass, physiological state), we found that the relative toxicity of IMI was significantly greater than for THX and CLO in red-winged blackbirds. This is similar to what has been observed in other bird species. For example, in eared doves, the median lethal dose (LD_50_) was 59 mg IMI/kg bw, 4248 mg CLO/kg bw, and 4366 mg THX/kg bw.^31^ Industry studies in bobwhite quail () reported LD50s at least ten times lower for IMI compared to CLO and THX (152 mg IMI/kg bw, >2000 mg CLO/kg bw, and 1552 mg THX/kg bw).^58^

In addition to large variation in the relative toxicity of different neonicotinoid compounds, there can also be large variation in species sensitivity. For example, for IMI, reported avian LD50s range from 14 mg/kg bw in grey partridge () to 283 mg/kg bw for mallards ().^53^ While our study was focused on sublethal effects and did not establish an LD_50_, we found evidence that red-winged blackbirds are moderately sensitive, with 20% mortality in the 40 mg IMI/kg bw dose group. Based on effects on mass, red-winged blackbirds are less sensitive to IMI than some other passerines. In white-crowned sparrows, a single dose of 3.9 mg/kg decreased body mass by 6% and extended stopover duration in migrating birds by a median of 3.5 days,^29^ whereas in the present study, 10 mg/kg bw had no effect on red-winged blackbird body mass, and 30 mg/kg bw decreased mass by 4.6%. When compared to another passerine species, the grayish baywings, red-winged blackbirds appear to have a similar sensitivity to IMI. Baywings exhibited the first symptoms of neurotoxicity at 20.6 mg/kg bw, with no effects at 8.48 mg/kg bw, and an LD_50_ of 57.11 mg/kg bw.^5^ Red-winged blackbirds had no or mild symptoms of neurotoxicity at 10 mg/kg and increasingly severe symptoms at 20 and 30 mg/kg bw. These differences in relative compound toxicity and species sensitivity could be related to several factors, including differences in binding affinity to nAChR, modulation of the cholinergic system, and differential P450 enzyme-mediated biotransformation potential among substances and species.^21,59–61^ Assessing sensitivity in additional wildlife species that are at risk of exposure to neonicotinoids would be beneficial for establishing ecologically relevant effects thresholds.

While this study was limited to a single acute exposure, chronic longer-term exposure to lower concentrations could have additive effects, particularly when mass loss is sustained. There is increasing evidence from field data that shows widespread exposures in wild bird populations,^16,17,62^ which suggests chronic effects are likely. These include disruption of organ function, immune function, oxidative stress, reproduction, and glucose metabolism.^4,53^ Chronic or subchronic exposure studies in rats and mice suggest organ system toxicity and altered organismal processes related to energy and metabolism. For example, chronic IMI exposure in mice (below reported NOAEL, 0, 0.5, 1.67, 5 mg/kg bw/day for 70 days) caused an imbalance in gut microbiota, functional liver damage, and disrupted bile acid production.^63^ Longer-term IMI exposure in rats (0.5 and 1.0 mg/kg bw/day for 60 days) damaged pancreatic tissue and resulted in a hyperglycemic affect.^64^ After a 90 day IMI (4 mg/kg bw/day) or CLO (12 mg/kg bw/day) exposure, fatty acids and total cholesterol levels increased in kidney tissue.^65^ Emerging evidence in birds indicates similar impairment of immune function, oxidative damage, and reproductive function linked to organ damage when chronically or subchronically exposed.^10,38,66,67^

While the toxicity of neonicotinoids will depend on the active ingredient, species sensitivity, and exposure duration, researchers should be aware that neonicotinoids exert consistent and repeatable effects of appetite suppression, followed by reduced and sometimes prolonged reduction in food consumption that can lead to changes in physiological state and concomitant mass loss. Food consumption, mass loss, and physiological state are less overt effects compared to visible symptoms of intoxication; however, when they are tracked, they are routinely documented and are often observed at lower concentrations. Effects on food consumption and body mass may also be longer lasting. In our study, birds exposed to the high dose of IMI had not fully recovered to predosing food consumption rates and body mass by 48 h, whereas by 24 h, there were no longer visible intoxication symptoms. Sublethal effects on appetite and condition can have subsequent effects on migration, reproduction, and survival^29,54,68^ and could be a mechanistic link between insecticide exposure and population-level effects.^69^

## Supplementary Material



## Notes

Detailed neurobehavioral severity scoring system, summary statistics for body mass and food consumption, summary statistics for fattening index and uric acid, and change in food consumption and body mass at 24 and 48 h postdosing (PDF)

## §

Science and Technology Branch, Environment and Climate change Canada, Dartmouth, Nova Scotia, Canada, B2Y 2N6
