# Supplementary material for: Comparative Sublethal Toxicity of Three Neonicotinoids in Red-Winged Blackbirds
Source: Environ Sci Technol. 2025 Jul 7;59(28):14268–78. doi: 10.1021/acs.est.5c03152 (PMC12288055; doi:10.1021/acs.est.5c03152)
Supplement: Supplementary file 1 [file es5c03152_si_001.pdf]

Comparative Sublethal Toxicity of Three Neonicotinoids in Red-winged Blackbirds

Margaret L. Eng[1,3] and Christy A. Morrissey[2]*

[1]Toxicology Centre, University of Saskatchewan, Saskatoon, S7N 5B3, Canada.

[2]Department of Biology, University of Saskatchewan, Saskatoon, S7N 5E2, Canada.

[3]current address: Science and Technology Branch, Environment and Climate change Canada, Dartmouth, Nova Scotia, Canada, B2Y 2N6

*Corresponding author: margaret.eng@ec.gc.ca

Supporting Information

- Table S1: Detailed neurobehavioural severity scoring system
- Table S2: Summary statistics for body mass and food consumption
- Table S3: Summary statistics for fattening index and uric acid
- Figure S1: Change in food consumption and body mass at 24- and 48-hours post-dosing

Table S1. Scoring system used to classify severity of neurobehavioural abnormalities in red-winged blackbirds following oral dosing with neonicotinoids. Each behavior category was scored from 0 to 5, and an overall score was calculated based on the average of all categories.

	Score					
	**0**	**1**	**2**	**3**	**4**	**5**
**Behavior Category**	**Normal, active**	**slight, indefinite signs**	**mild depression**	**moderate depression**	**Severely depressed**	**Virtual paralysis**
**Activity**	Active, hopping	Slow to move, resting frequently	Lethargic, but still responsive to environment	Lethargy/sleepiness, usually sitting, reluctant to stand, move	Not moving, may have tremors	Immobile, tremors
**Balance**	Good balance	Maneuvers to maintain balance	Unstable, uncoordinated, falling off perch, misses perch	Does not perch, rests on ground, struggles to stand	Severe impairment of ability to stand, walk, fly	Unable to stand, move
**Feeding**	Forages, drinks, preens	Slow to feed, may have unpreened appearance	Infrequent feeding, may have fluffed feathers, unpreened appearance	Little to no foraging, fluffed feathers, drooped wings	Not eating, fluffed feathers, drooped wings	Not eating, fluffed feathers, drooped wings
**Response**	Moves away when approached	Moves away when approached	Moves away when approached, slower, easier to catch	Hesitates to move away, may stumble, slow	Minimally responsive, unable to move away when approached	Does not react when approached
**Respiration**	No respiratory difficulty	Mild or no respiratory difficulty, may have slowing breathing	Slight panting, slower breathing	Mildly increased respiratory effort, slow breathing	Respiratory difficulty, slow breathing, open mouth breathing	Respiratory difficulty, very slow breathing, open mouth breathing

Table S2. Body mass (g) and food consumption (g food/g bw/day) in female (F) and male (M) red-winged blackbirds immediately before (pre) dosing with imidacloprid (IMI), clothianidin (CLO), or thiamethoxam (THX), and again at 24-hours and 48-hours post-dosing, as well as before food restriction, after 24-hours of food restriction, and at 48-hours after restriction started (after being returned to ad libitum food for 24-hours).

		Mass (g)				Consumption (g food/g bw/day)			
		F		M		F		M	
Treatment	Time	N	Mean (SE)	N	Mean (SE)	N	Mean (SE)	N	Mean (SE)
Control	pre	8	45.95 (0.87)	7	70.69 (1.96)	8	0.33 (0.02)	7	0.30 (0.04)
	24-hr post	8	45.60 (0.98)	7	71.06 (1.87)	8	0.33 (0.01)	7	0.28 (0.03)
	48-hr post	8	46.08 (0.90)	7	70.93 (1.73)	8	0.36 (0.04)	7	0.31 (0.03)
IMI Low	pre	4	42.55 (0.35)	5	64.38 (2.08)	4	0.51 (0.02)	5	0.25 (0.01)
	24-hr post	4	42.30 (0.25)	5	64.78 (1.86)	4	0.32 (0.04)	5	0.25 (0.03)
	48-hr post	4	43.35 (0.64)	5	64.84 (1.78)	4	0.35 (0.03)	5	0.23 (0.00)
IMI Med	pre	4	45.17 (0.55)	6	69.32 (0.51)	4	0.32 (0.04)	6	0.34 (0.02)
	24-hr post	4	44.60 (0.47)	6	68.12 (1.10)	4	0.30 (0.05)	6	0.21 (0.01)
	48-hr post	4	45.98 (0.66)	6	69.38 (0.93)	4	0.43 (0.04)	6	0.33 (0.00)
IMI High	pre	4	45.17 (0.70)	6	67.07 (0.54)	4	0.55 (0.06)	6	0.31 (0.03)
	24-hr post	4	43.30 (0.95)	4	63.27 (0.99)	4	0.14 (0.03)	4	0.11 (0.02)
	48-hr post	4	44.62 (0.71)	4	65.78 (0.63)	4	0.38 (0.04)	4	0.32 (0.05)
CLO Low	pre	4	46.42 (0.93)	6	67.38 (1.08)	4	0.32 (0.02)	4	0.24 (0.04)
	24-hr post	4	46.35 (0.51)	6	67.73 (0.94)	4	0.31 (0.01)	6	0.22 (0.02)
	48-hr post	4	46.60 (0.53)	6	67.37 (0.78)	4	0.42 (0.01)	6	0.27 (0.02)
CLO Med	pre	4	45.33 (0.69)	6	68.37 (1.54)	4	0.33 (0.02)	6	0.27 (0.02)
	24-hr post	4	45.52 (0.57)	6	68.93 (1.56)	4	0.29 (0.02)	6	0.27 (0.02)
	48-hr post	4	44.88 (0.69)	6	68.87 (1.47)	4	0.38 (0.01)	6	0.29 (0.01)
CLO High	pre	4	46.33 (1.10)	5	67.46 (1.44)	4	0.31 (0.02)	3	0.23 (0.01)
	24-hr post	4	46.22 (1.09)	5	68.58 (1.59)	4	0.25 (0.01)	3	0.20 (0.03)
	48-hr post	4	46.27 (1.35)	5	68.12 (1.30)	4	0.33 (0.01)	3	0.28 (0.06)
THX Low	pre	4	46.15 (1.23)	6	67.78 (1.59)	4	0.32 (0.01)	6	0.26 (0.02)
	24-hr post	4	46.00 (0.97)	6	68.02 (1.39)	4	0.26 (0.02)	6	0.23 (0.02)
	48-hr post	4	45.88 (0.91)	6	69.15 (1.57)	4	0.29 (0.03)	6	0.25 (0.01)
THX Med	pre	6	45.73 (0.74)	4	68.10 (1.33)	6	0.34 (0.01)	4	0.31 (0.02)
	24-hr post	6	45.12 (0.73)	4	68.30 (1.65)	6	0.27 (0.00)	4	0.29 (0.01)
	48-hr post	6	45.68 (0.69)	4	69.62 (1.41)	6	0.34 (0.02)	4	0.28 (0.01)
THX High	pre	4	46.12 (0.69)	6	69.30 (1.64)	4	0.33 (0.02)	6	0.24 (0.01)
	24-hr post	4	45.35 (0.67)	6	68.98 (1.49)	4	0.29 (0.01)	6	0.23 (0.01)
	48-hr post	4	46.08 (0.68)	6	69.63 (1.44)	4	0.37 (0.04)	6	0.24 (0.01)
Moderate Food Restriction	pre	3	46.17 (3.20)	6	70.75 (3.09)	3	0.63 (0.03)	6	0.34 (0.03)
	24-hr post	3	44.07 (2.74)	6	68.97 (2.81)	3	0.25 (0.01)	6	0.17 (0.02)
	48-hr post	3	45.17 (3.30)	6	70.38 (2.70)	3	0.67 (0.03)	6	0.36 (0.03)
High Food Restriction	pre	5	47.02 (1.55)	4	68.40 (1.79)	5	0.60 (0.01)	4	0.38 (0.02)
	24-hr post	5	44.48 (1.57)	4	64.28 (1.75)	5	0.12 (0.01)	4	0.13 (0.01)
	48-hr post	5	46.00 (1.56)	4	66.55 (1.62)	5	0.65 (0.02)	4	0.44 (0.03)

Table S3. Fattening Index score and plasma Uric Acid (mM) in female (F) and male (M) red-winged blackbirds. Blood samples were taken before (pre) dosing with imidacloprid (IMI), clothianidin (CLO), thiamethoxam (THX), or food restrictions started, and after (post). Blood samples were taken 24-hours apart, at 18-hours pre-dosing, and at the same time the next day at 6-hours post-dosing.

		Fattening Index				Uric Acid (m*M*)			
		F		M		F		M	
Treatment	Time	N	Mean (SE)	N	Mean (SE)	N	Mean (SE)	N	Mean (SE)
Control	pre	8	0.11 (0.18)	7	0.23 (0.31)	8	0.94 (0.15)	7	0.97 (0.12)
	post	8	0.01 (0.31)	7	-0.18 (0.41)	8	0.90 (0.11)	7	0.86 (0.13)
IMI Low	pre	4	0.50 (0.27)	5	0.09 (0.28)	4	1.14 (0.36)	5	0.95 (0.22)
	post	4	0.60 (0.25)	5	-0.71 (0.17)	4	0.69 (0.21)	5	0.98 (0.34)
IMI Med	pre	4	0.25 (0.40)	6	0.25 (0.36)	4	0.73 (0.14)	6	1.06 (0.14)
	post	4	-1.72 (0.36)	6	-1.35 (0.67)	4	0.84 (0.21)	6	0.79 (0.18)
IMI High	pre	4	0.67 (0.18)	6	0.16 (0.16)	4	0.69 (0.36)	6	0.78 (0.11)
	post	4	-1.90 (0.67)	4	-2.51 (0.41)	4	0.81 (0.29)	4	1.03 (0.17)
CLO Low	pre	4	0.71 (0.46)	6	0.33 (0.30)	4	1.20 (0.09)	6	0.95 (0.12)
	post	4	0.46 (0.46)	6	-0.08 (0.46)	4	1.19 (0.11)	6	0.93 (0.11)
CLO Med	pre	4	0.28 (0.37)	6	0.17 (0.30)	4	1.13 (0.16)	6	0.98 (0.07)
	post	4	-0.30 (0.12)	6	-0.55 (0.29)	4	1.11 (0.12)	6	0.89 (0.05)
CLO High	pre	4	0.17 (0.67)	5	0.09 (0.25)	4	1.01 (0.19)	5	0.94 (0.04)
	post	4	-0.60 (0.36)	5	-0.27 (0.52)	4	0.96 (0.06)	5	1.31 (0.21)
THX Low	pre	4	0.11 (0.21)	6	0.22 (0.25)	4	1.13 (0.14)	6	1.04 (0.18)
	post	4	0.22 (0.14)	6	-0.23 (0.35)	4	0.85 (0.15)	6	0.82 (0.07)
THX Med	pre	6	-0.65 (0.20)	4	0.28 (0.14)	6	1.57 (0.13)	4	0.74 (0.11)
	post	6	-0.70 (0.16)	4	0.41 (0.21)	6	0.73 (0.09)	4	0.67 (0.05)
THX High	pre	4	0.18 (0.19)	6	-0.01 (0.48)	4	1.22 (0.12)	6	1.18 (0.22)
	post	4	0.39 (0.17)	6	-0.34 (0.19)	4	0.85 (0.05)	6	1.00 (0.20)
Moderate Food	pre	3	1.31 (0.54)	6	1.38 (0.19)	3	0.85 (0.30)	6	0.72 (0.09)
Restriction	post	3	0.34 (0.27)	6	0.51 (0.28)	3	0.81 (0.21)	6	0.79 (0.11)
High Food	pre	5	1.46 (0.22)	4	1.21 (0.36)	5	0.82 (0.08)	4	0.62 (0.08)
Restriction	post	5	-0.08 (0.30)	4	-0.37 (0.42)	5	0.94 (0.21)	4	1.00 (0.20)

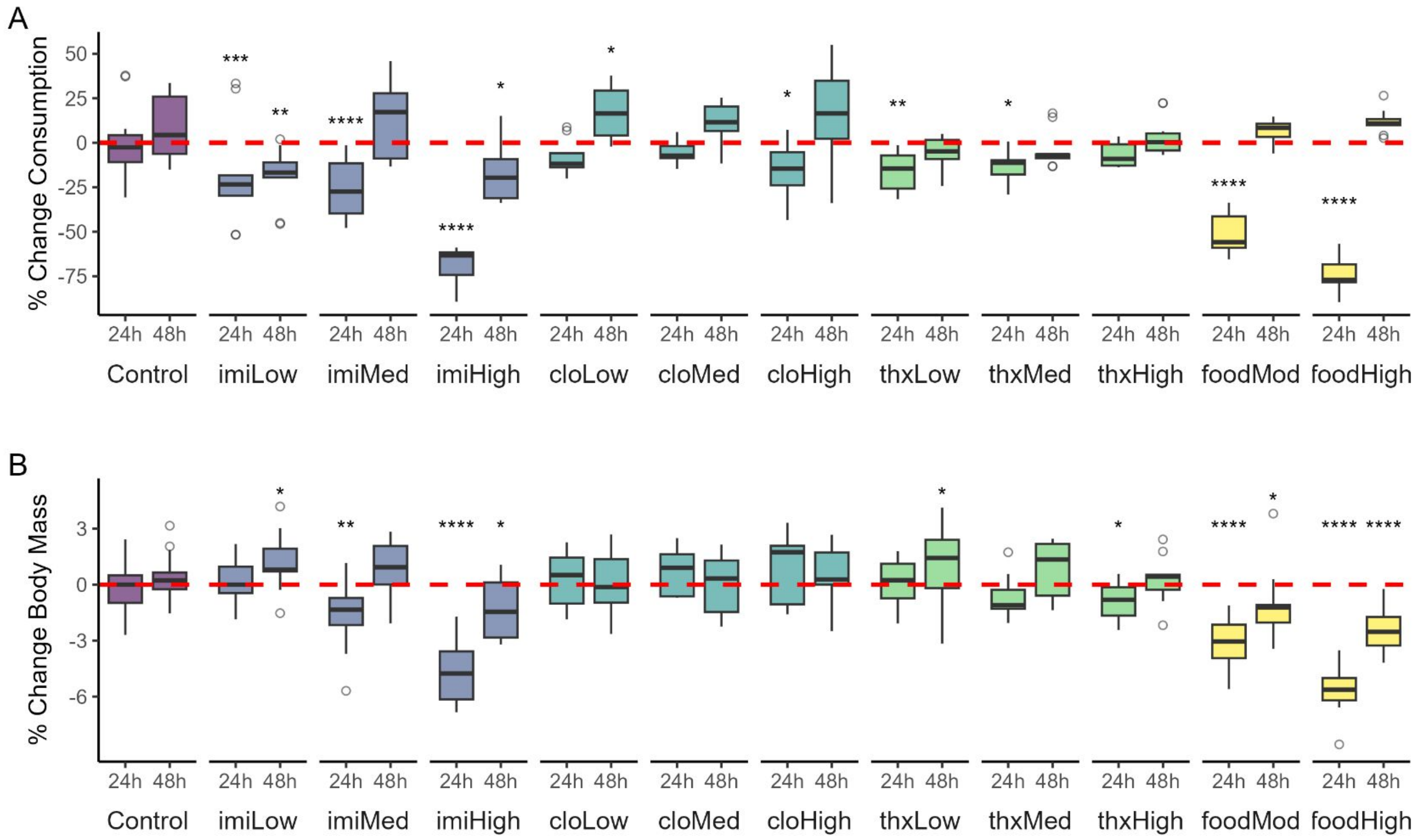

Figure S1. Percent change in (A) daily food consumption and (B) body mass between pre-dosing and post-dosing in red-winged blackbirds exposed to a vehicle control (sunflower oil), imidacloprid (IMI), clothianidin (CLO), thiamethoxam (THX), or food restriction (moderate or high). Changes are presented for measures taken 24- and 48-hours post-dosing compared to immediately prior to dosing. Asterisks indicate significant difference between pre-dosing and post-dosing measures within a treatment group (* $p < 0.05$ ** $p < 0.01$ *** $p < 0.001$ **** $p < 0.0001$). Boxes indicate interquartile range (IQR), middle lines indicate median, whiskers show the minimum and maximum values within 1.5x IQR, and circles represent outliers (>1.5x IQR from box).
